# Successful Application of Artificial Intelligence‐Assisted Analysis of Invasive Pulmonary Adenocarcinoma Less Than 6 mm in Size: A Case Report and Literature Review

**DOI:** 10.1111/crj.70073

**Published:** 2025-05-28

**Authors:** Lu Zhang, Dawei Yang, Xianwei Ye, Chunxue Bai

**Affiliations:** ^1^ Department of Respiratory and Critical Care Medicine Guizhou Provincial People's Hospital Guiyang China; ^2^ Department of Respiratory and Critical Care Medicine Fudan University Affiliated Zhongshan Hospital Shanghai China; ^3^ Shanghai Respiratory Internet of Things Medical Engineering Technology Research Center Shanghai China

**Keywords:** artificial intelligence, case report, invasive pulmonary adenocarcinoma, lung nodule

## Abstract

**Introduction:**

Screening of lung nodules helps on early diagnosis of lung cancer, especially invasive pulmonary adenocarcinoma. Artificial intelligence (AI) has been applied in diagnosis of cancers. We used the AI‐assisted lung nodule diagnostic system in the screening of lung nodules and lung cancer.

**Case Presentation:**

A 66‐year‐old male complained of coughs and nodules in the right lung of 3‐year duration. A ground‐glass opacity was found in the right upper lung by routine computed tomography (CT). He had no family history of cancer, genetic diseases, or infectious diseases. AI‐assisted analysis found four nodules, of which one was with the risk of malignancy of 88% (LungRads3), one was with the risk of malignancy of 15% (LungRads2), and the other two were smaller in size and considered benign. The patient underwent a thoracoscopic wedge resection of the right upper lung. The intraoperative frozen section pathology report confirmed invasive pulmonary adenocarcinoma, grade II, and primarily of alveolar and adherent types without metastasis.

**Conclusion:**

In summary, AI‐assisted lung nodule diagnostic system is effective in the screening of lung nodules and the differentiation between benign and malignant.

## Graphical abstract

A 66‐year‐old male complained of coughs and nodules in the right lung of 3‐year duration. AI‐assisted lung nodule diagnostic system help confirmed the patient of invasive pulmonary adenocarcinoma, grade II, and primarily of alveolar and adherent types without metastasis.

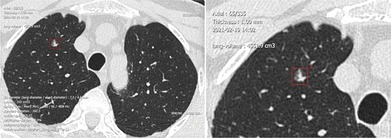

## 1 Introduction

Lung cancer is one of the most common cancers and a leading cause of cancer‐related deaths. Lung cancer is usually asymptomatic at an early stage and often diagnosed at a late stage, which explains the high mortality rate [1, 2]. Early screening and diagnosis can dramatically decrease the mortality associated with lung cancer [1, 2]. Low‐dose computed tomography (LDCT) is an effective early screening tool for lung cancer for the purpose of reducing mortality [2, 3]. The finding of lung nodules is a common finding during lung cancer screening. Most lung nodules are benign, especially the small nodules [4]. Early screening for lung nodules is of particular importance for diagnosing invasive pulmonary adenocarcinoma [5]. Radiologists are expected to correctly identify all lung nodules and determine the attributes (solid, partially solid, or nonsolid) by examining the boundaries, shape, location, and size [4]. This process may be highly challenging and time‐consuming, and one way to overcome this problem is artificial intelligence (AI) [6]. An AI‐assisted lung nodule diagnostic system (hereafter referred to as an AI‐assisted system) has demonstrated good diagnostic performance for lung nodules and lung cancers according to the current experience from clinical applications [6, 7]. Herein, we report a patient who received an AI‐assisted differential diagnosis of lung nodules. The AI‐assisted system successfully detected invasive pulmonary adenocarcinoma < 6 mm in size.

## 2 Case Description

### 2.1 Patient Specific Information

**FIGURE 1:**
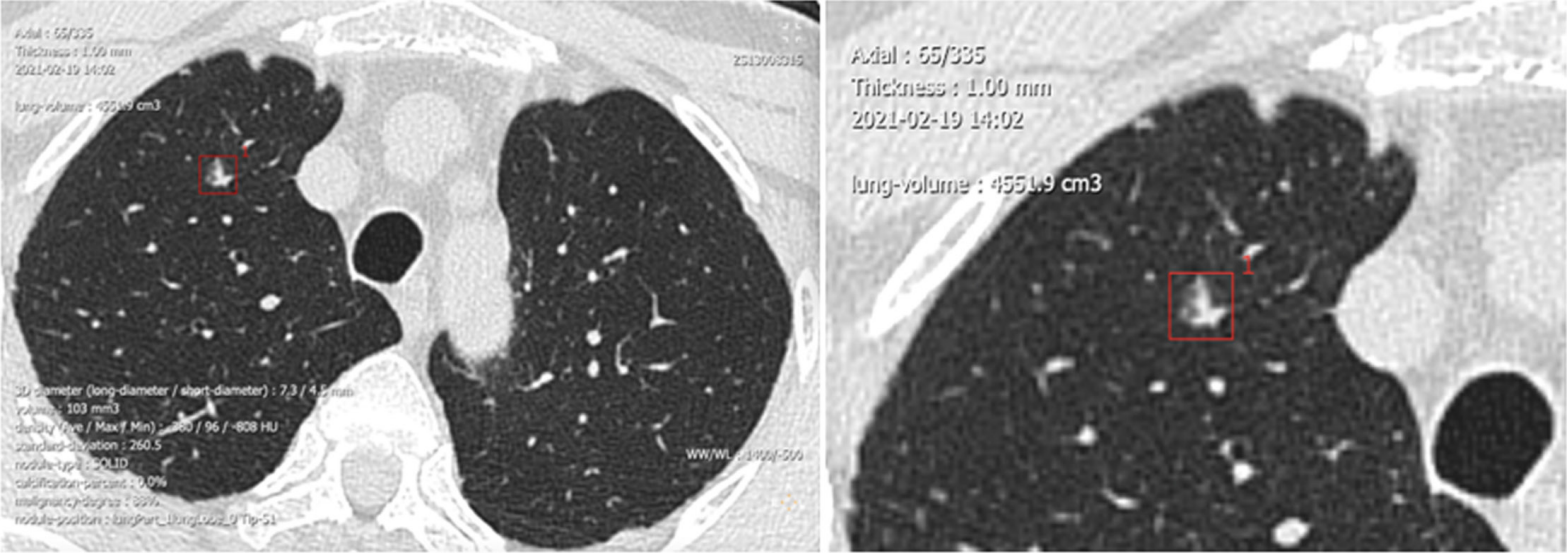
A ground‐glass opacity found in the right upper lung by routine computed tomography (CT).

A 66‐year‐old male sought evaluation at our hospital on February 10, 2021 for a cough and nodules in the right lung of 3‐year duration. A routine chest computed tomography (CT) (320 row 640‐slice ultrafast spiral CT, Siemens, Germany) after admission demonstrated the following: a ground‐glass opacity (GGO) in the right upper lung with a major diameter of approximately 6 mm (Figure 1) and a nonuniform density. There was a small strip shadow in the right lung (im18), with small cystic lesions scattered in both lungs. No bronchial lumen obstruction was noted. There were no enlarged lymph nodes in the lung hila or the mediastinum. The pleura was not thickened, and there were no pleural effusions. Compared with previous chest CT results from another hospital presented by the patient, the nodules were morphologically changed, and the patient's nodules showed burr and vacuole signs, which suggested a high possibility of malignancy.

The patient had a clear conscious, lucidity, steady breathing, and a normal body habitus upon pre‐admission physical examination. No superficial lymph nodes were enlarged nor was the thyroid gland increased in size. There were no thoracic deformities. The bilateral lung sounds were clear upon percussion. The breathing sounds were clear upon auscultation. B‐mode scans were performed in the liver, spleen, portal vein, pancreas, kidneys, adrenal glands, ureters, cervical mass, and lymph nodes. Liver cysts were visualized. Conventional transthoracic echocardiography (color Doppler echocardiography + left cardiac function measurement + tissue Doppler imaging [TDI]) detected no abnormalities in the resting state. Conventional ECG showed a sinus rhythm.

#### 2.1.1 History of Past Illnesses and Family History

In 2016, the patient underwent a laparoscopic radical prostatectomy + pelvic adhesiolysis + bladder neck reconstruction in our hospital. The postoperative follow‐up evaluation in 2016 after the surgery revealed nodules in the right upper lung. Subsequently the patient had a productive cough, but no chest pain, hemoptysis, chest tightness, or polypnea. But he did not come to our hospital for further treatment. The patient had not history of hypertension, diabetes, hepatitis, tuberculosis, or trauma. The patient had no history of food or drug allergies. He did not smoke cigarettes or consume excessive amounts of alcohol. The patient did not reside in an endemic area. There was no family history of cancer or genetic diseases. There was no family history of infectious diseases. The patient's children were in good physical health.

#### 2.1.2 AI‐Assisted Auxiliary Examinations

**FIGURE 2:**
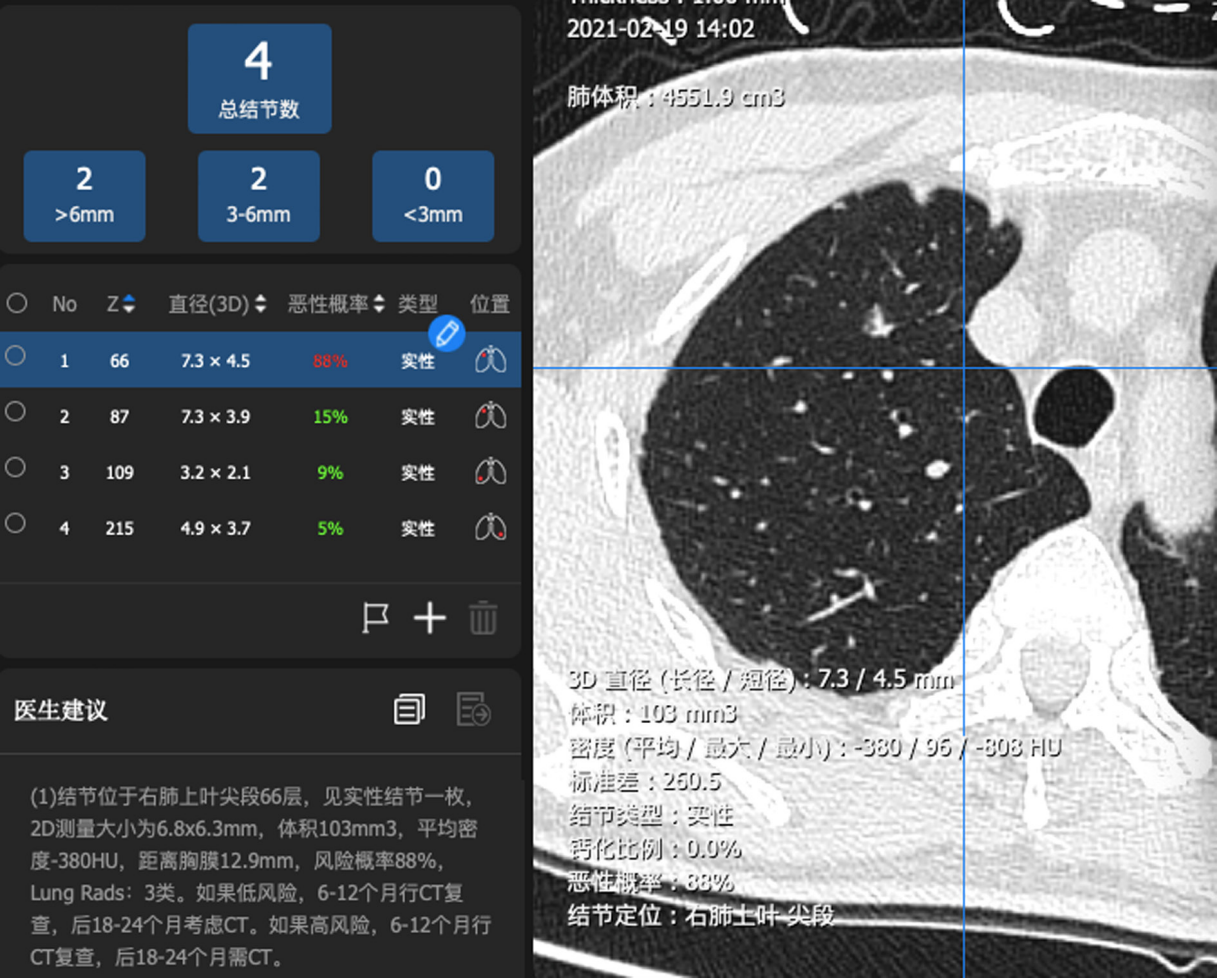
One solid nodule with the diameter of 7.3 mm in the upper lobe of the right lung (66/335). Risk of malignancy:88% (LungRads3).

The patient underwent AI‐assisted analysis. There were four nodules in the lung. One solid nodule was demonstrated in the upper lobe of the right lung (66/335; Figure 2). The longest diameter on the 3D image was 7.3 mm, the volume was 103 mm^3^, and the average density was −380 HU. There were no central calcifications, and the risk of malignancy was 88%, which was a high level (LungRads3). One solid nodule was located in the upper lobe of the right lung (87/335). The longest diameter on the 3D image was 7.3 mm, the volume was 60.3 mm^3^, and the average density was −302.4 HU. There were no central calcifications. The risk of malignancy was 15%, which was a low level (LungRads2). The remaining two nodules were smaller in size and considered benign. Laboratory testing showed three circulating genetically abnormal cells. The levels of CEA, SCC, CYFRA21‐1, NSE, and ProGRP were normal.

The AI system is used only in our hospital. It is not a commercial product, and it is not available for general use now. The AI system is developed by the Professor Chunxue Bai and his team and is developed based on more than 10 years of technology and the big data of clinical data. Each time, the system is started, and the data are read; the AI system needs to be tested again. The system will update and correct based on each data adding into the system.

#### 2.1.3 Diagnosis and Treatment

**FIGURE 3:**
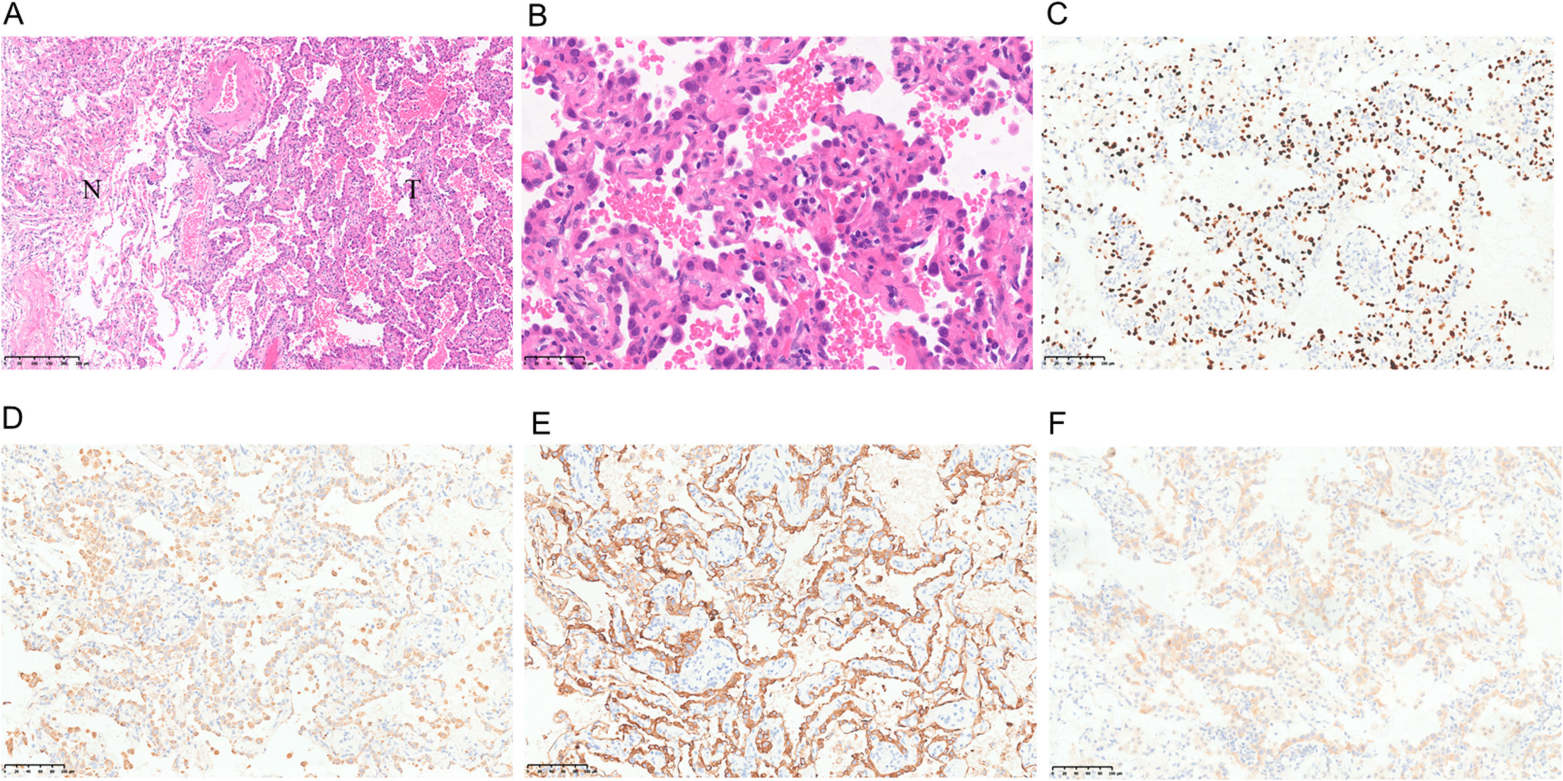
Pathological result. (A,B) HE staining of tumor and surrounding lung; (C) TTF‐1(+); (D) Napsin A(+); (E) CK7(+); (F) EGFR L858.

A high‐resolution CT scan of the lungs combined with AI‐assisted analysis identified several nodules in the lung, which had a high risk of malignancy. A right superior nodulectomy was recommended. On March 25, 2021, the patient underwent a thoracoscopic wedge resection of the right upper lung under composite anesthesia. Preoperative pulmonary function tests were performed. The nodules were marked before surgery by a hook. A 4‐cm operative incision was made in the right fifth intercostal space along the anterior axillary line. A 1.5‐cm observation incision was made in the right eighth intercostal space along the anterior axillary line. The pleural cavity was inspected for adhesions. There were no pleural effusions or nodules. A nodule was noted in the right upper lung. A mass was observed 0.9 cm from the resection margin and within 0.5 cm of the pleura. The largest diameter of this mass was 0.6 cm. The cross‐section was gray–white in color, with a solid texture and clearly defined boundaries. A wedge resection of the right upper lung was performed using a straight cutter stapler. The wedge‐shaped area was 11 × 4.6 × 2.4 cm in size. The operation was completed successfully, and the patient did not require a blood transfusion. Symptomatic and supportive treatment was provided postoperatively. The intraoperative frozen section pathology report was as follows: right upper lung nodule, invasive pulmonary adenocarcinoma (an invasive lesion approximately 6 mm in size), grade II, and primarily of alveolar and adherent types; and the tumor did not involve the visceral pleura. Lymph node group numbers 2, 4, 7, and 10 were submitted for postoperative macroscopic examination. One lymph node from group number 2 was submitted for evaluation after pulmonary malignant tumor; no metastasis was detected (0/1). Three lymph nodes from group number 4 were submitted, and no metastases were detected (0/3). One lymph node from group number 7 was submitted, and no metastasis was detected (0/1). Three lymph nodes from group number 10 were submitted, and no metastases were detected (0/3). The immunohistochemical results are shown in Figure 3. Molecular pathology genetic testing showed that the tumor cells were suitable for testing. There was an exon‐21 L858R mutation in the EGFR gene, but no mutations were detected in exons 18, 19, and 21. The final diagnosis of the right upper lung nodule was confirmed as invasive adenocarcinoma (IAC).

#### 2.1.4 Follow‐up

The patient was discharged on March 29, 2021 with no medications according to treatment guidelines. He was advised to have dynamic chest high‐resolution CT every 3 months. The patient had a re‐examination chest CT scan at the Hospital on August 9, 2021, which indicated typical changes in the right lung. There were small nodules in both lungs, which were suspected to be inflammatory. Oral levofloxacin hydrochloride was given as anti‐infective therapy.

## 3 Ethics Approval and Consent to Participate

This study was conducted following the Helsinki Declaration and was approved by the ethical committee of our hospital. The written informed consent was obtained from the patient to publish the related data and images of this case.

## 4 Discussion

There is increased awareness of the importance of periodic medical evaluations in recent years. In addition, low‐dose chest CT has become more widely accepted. The number of cases diagnosed with lung cancers < 1 cm in diameter has increased for the above reasons [8]. Lung cancers < 1 cm in diameter, also known as sub‐centimeter lung cancers, usually have complex radiographic features, differentiated situation, and highly differential prognosis. The therapeutic intervention for lung cancers generally varies among patients [9]. According to the guidelines and consensus on the diagnosis and treatment of lung nodules [10, 11], the nature of lung nodules < 1 cm in size is challenging due to the small size of the biopsy. Thus, observation is usually recommended for these nodules, and the patients are advised to undergo follow‐up re‐examinations every 3–6 months. The patient reported herein was re‐examined after discharge, which was consistent with evidence‐based medicine.

### 4.1 CT Imaging of Lung Tumors and Pathologic Types

Pulmonary adenocarcinomas account for > 50% of early‐stage lung cancers [5]. Microinvasive adenocarcinoma (MIA) refers to a single mass with the largest diameter ≤ 3 cm in which the tumor cells grow with complete adherence to the alveolar walls, accompanied by an invasive lesion with a maximum diameter ≤ 5 mm. When the invasive lesion has a maximum diameter > 5 mm, the adenocarcinoma is an IAC, primarily of the adherent type. Pulmonary adenocarcinoma usually presents as a subsolid nodule (SN) on CT scan. Successive stages of development of pulmonary adenocarcinoma have been identified as follows: preinvasive lesion, including atypical adenomatous hyperplasia (AAH) and adenocarcinoma in situ (AIS) [12], MIA, and IAC [5]. The solid components in the lung tumors show massive, aggregate growth of fibrous scars or tumor cells. The ratio of GGO‐to‐solid components can represent the degree of malignancy. A study has shown that among all sub‐centimeter nodules, GGO nodules are more likely to develop into cancers than solid nodules during a 2‐year follow‐up [13]. In one cohort study, > 55% of the sub‐centimeter GGO nodules contained progressively invasive lesions [14]. In our case, the nodule in the right upper lung was 6 mm in size. The CT scan indicated GGO, a typical radiographic feature of sub‐centimeter lung cancer. The proportion of GGO components decreases from AAH‐to‐IAC, while that of solid components increases. The higher the degree of malignancy, the worse the prognosis [15]. In the current case, the proportion of GGO components decreased, while solid components increased. In accition, radiographic features of malignancy were observed, and the risk of malignancy was considered high. The AI‐assisted analysis identified a high‐risk nodule in this case. When combined with the pathology results, the nodule was confirmed to be a sub‐centimeter IAC.

### 4.2 Relationship Between Tumor Size and Pathology

According to the 2021 WHO classification system of lung adenocarcinoma, AAH usually has a size < 0.5 cm, while AIS and MIA have a size < 3 cm. Lampen‐Sachark et al. [16] highlighted a significant difference in tumor diameter measured on CT images and surgically resected gross samples. The former is generally larger than the latter. The average difference has been estimated to be 5.49 mm after a review of measurement data. According to relevant data [17], the size of AAH ranged from 6.0 to 8.0 mm, with a peak of 7.8 mm. The size of MIA ranges from 7.5 to 12.5 mm, with a peak of 11.8 mm. The size of IAC ranges from 20.0 to 25.0 mm, with a peak of 23.1 mm. As shown by the ROC curves, the cutoff value of the lesion size differentiating IAC from AAH and MIA is 15.35 mm. The sensitivity was 80.8%, and the specificity was 90.4%. Zhan et al. [18] reported that CT scans can differentiate IAC, AIS, and MIA for a GGO 5–10 mm in size. GGOs > 8.12 mm with −449.52 HU are more likely to be an IAC [18]. In our case, the AI‐assisted analysis reported a maximum diameter of 7.4 mm for the nodule in the right upper lung on the 3D image, which was > 6 mm on the planar image. Because the former could more accurately characterize the lung nodules, we finally adopted a diameter measured by the AI‐assisted analysis.

### 4.3 AI‐Assisted Analysis Combined With Circulating Genetically Abnormal Cells Test to Achieve Precision Treatment

Liquid biopsy has the benefits of noninvasiveness and ease of application, popularization, and continuous monitoring. Lung cancer screening and early diagnosis are the two most important application directions of liquid biopsy [19]. For small lung nodules (< 8 mm) that are difficult to diagnose based on pathologic findings and radiography, a liquid biopsy can be used to detect DNA released by the tumors to inform patient care. Bai et al. [20] suggested that the AI‐assisted technique and the circulating genetically abnormal cells play vital roles in the early detection of early‐stage lung cancer. Both methods also help predict tumor recurrence and differentiate benign tumors from malignant tumors [20]. An AI‐assisted analysis requires using DICOM files with a slice thickness of 1 mm, which carries more information for the accurate differentiation between benign and malignant lung nodules. Our reported case had AI‐assisted analysis and circulating genetically abnormal cell testing early, which effectively prevented tumor progression.

## 5 Conclusions

In summary, AI‐assisted lung nodule diagnostic system can effectively screen lung nodules and differentiate benign from malignant lung nodules.

## Author Contributions

Lu Zhang and Chunxue Bai conceived and designed this study. Lu Zhang, Dawei Yang, Xianwei Ye, and Chunxue Bai participated in the clinical case research and data collection, as well as drafting the manuscript. All authors read and approved the final manuscript.

## Ethics Statement

The authors were accountable for all aspects of the work in ensuring that questions related to the accuracy or integrity of any part of the work were appropriately investigated and resolved. All procedures performed in this study were by the ethical standards of the institutional and/or national research committee(s) and with the Helsinki Declaration (as revised in 2013).

## 6 Consent

Publication of this case report and accompanying images was waived from patient consent according to Guizhou Provincal People's Hospital ethics committee review board.

## Conflicts of Interest

The authors declare no competing interests.

## Data Availability

The datasets used during the current study are available from the corresponding author on reasonable request.

## References

[1] N. Becker , E. Motsch , A. Trotter , et al., “Lung cancer Mortality Reduction by LDCT Screening‐Results From the Randomized German LUSI Trial,” International Journal of Cancer 146, no. 6 (2020): 1503–1513. 31162856 10.1002/ijc.32486

[2] National Lung Screening Trial Research Team , “Lung Cancer Incidence and Mortality With Extended Follow‐Up in the National Lung Screening Trial,” Journal of Thoracic Oncology 14, no. 10 (2019): 1732–1742. 31260833 10.1016/j.jtho.2019.05.044 PMC6764895

[3] Y. Du , Y. Li , M. D. Dorrius , et al., “Comparison of National Comprehensive Cancer Network and European Position Statement Protocols for Nodule Management in Low‐Dose Computed Tomography Lung Cancer Screening in a General Chinese Population,” Journal of Thoracic Disease 13 (2021): 6855–6865. 35070370 10.21037/jtd-21-1312 PMC8743405

[4] P. J. Mazzone and L. Lam , “Evaluating the Patient With a Pulmonary Nodule: A Review,” Journal of the American Medical Association 327 (2022): 264–273. 35040882 10.1001/jama.2021.24287

[5] L. Succony , D. M. Rassl , A. P. Barker , F. McCaughan , and R. C. Rintoul , “Adenocarcinoma Spectrum Lesions of the Lung: Detection, Pathology and Treatment Strategies,” Cancer Treatment Reviews 99 (2021): 102237. 34182217 10.1016/j.ctrv.2021.102237

[6] J. Gong , J. Liu , W. Hao , et al., “A Deep Residual Learning Network for Predicting Lung Adenocarcinoma Manifesting as Ground‐Glass Nodule on CT Images,” European Radiology 30 (2020): 1847–1855. 31811427 10.1007/s00330-019-06533-w

[7] L. Li , Z. Liu , H. Huang , et al., “Evaluating the Performance of a Deep Learning‐Based Computer‐Aided Diagnosis (DL‐CAD) System for Detecting and Characterizing Lung Nodules: Comparison With the Performance of Double Reading by Radiologists,” Thoracic Cancer 10 (2019): 183–192. 30536611 10.1111/1759-7714.12931 PMC6360226

[8] M. Goto , K. Kawaguchi , T. Fukui , et al., “Verification of T Descriptor With Consolidation Size for Sub‐Centimeter Non‐Small Cell Lung Cancer,” Surgery Today 49 (2019): 907–912. 31115697 10.1007/s00595-019-01821-w

[9] S. C. Tsai , T. C. Wu , Y. L. Lai , and F. C. Lin , “Preoperative Computed Tomography‐Guided Pulmonary Nodule Localization Augmented by Laser Angle Guide Assembly,” Journal of Thoracic Disease 11 (2019): 4682–4692. 31903257 10.21037/jtd.2019.10.60 PMC6940224

[10] C. Bai , C. M. Choi , C. M. Chu , et al., “Evaluation of Pulmonary Nodules: Clinical Practice Consensus Guidelines for Asia,” Chest 150 (2016): 877–893. 26923625 10.1016/j.chest.2016.02.650

[11] Lung Cancer Group of Chinese Thoracic Society, Expert Group of China Lung Cancer Prevention and Control Alliance , “Chinese Expert Consensus on Diagnosis and Treatment of Pulmonary Nodules [2018 Edition],” Chinese Journal of Tuberculosis and Respiratory Medicine 2018, no. 41 (2019): 763–771.

[12] M. Peng , Z. Li , H. Hu , et al., “Pulmonary Ground‐Glass Nodules Diagnosis: Mean Change Rate of Peak CT Number as a Discriminative Factor of Pathology During a Follow‐Up,” British Journal of Radiology 89 (2016): 20150556. 26562098 10.1259/bjr.20150556 PMC4985203

[13] K. E. Shin , K. S. Lee , C. A. Yi , et al., “Subcentimeter Lung Nodules Stable for 2 Years at LDCT: Long‐Term Follow‐Up Using Volumetry,” Respirology 19 (2014): 921–928. 24934105 10.1111/resp.12337

[14] F. Wu , S. P. Tian , X. Jin , et al., “CT and Histopathologic Characteristics of Lung Adenocarcinoma With Pure Ground‐Glass Nodules 10 mm or Less in Diameter,” European Radiology 27 (2017): 4037–4043. 28386719 10.1007/s00330-017-4829-5

[15] M. Ikehara , H. Saito , T. Kondo , et al., “Comparison of Thin‐Section CT and Pathological Findings in Small Solid‐Density Type Pulmonary Adenocarcinoma: Prognostic Factors From CT Findings,” European Journal of Radiology 81 (2012): 189–194. 20965677 10.1016/j.ejrad.2010.09.026

[16] K. Lampen‐Sachar , B. Zhao , J. Zheng , et al., “Correlation Between Tumor Measurement on Computed Tomography and Resected Specimen Size in Lung Adenocarcinomas,” Lung Cancer 75 (2012): 332–335. 21890229 10.1016/j.lungcan.2011.08.001 PMC4441034

[17] L. Yang , Comparative Study Between CT Performance and IASLC/ATS/ERS New Classification Pathologic of Small Lung Adenocarcinoma With Ground‐Glass Opacity. (master’s thesis, Qingdao University, 2015).

[18] Y. Zhan , X. Peng , F. Shan , et al., “Attenuation and Morphologic Characteristics Distinguishing a Ground‐Glass Nodule Measuring 5–10 mm in Diameter as Invasive Lung Adenocarcinoma on Thin‐Slice CT,” AJR. American Journal of Roentgenology 213 (2019): W162–W170. 31216199 10.2214/AJR.18.21008

[19] X. D. Jiao , L. R. Ding , C. T. Zhang , et al., “Serum Tumor Markers for the Prediction of Concordance Between Genomic Profiles From Liquid and Tissue Biopsy in Patients With Advanced Lung Adenocarcinoma,” Translational Lung Cancer Research 10 (2021): 3236–3250. 34430361 10.21037/tlcr-21-543 PMC8350084

[20] N. Wang , Y. Yang , S. Chen , et al., “Progress of Liquid Biopsy Technique and Clinical Application in Lung Cancer,” Chinese Journal of Tuberculosis and Respiratory Diseases 41 (2018): 881–883. 30423633 10.3760/cma.j.issn.1001-0939.2018.11.011

